# Impact of rigid gas-permeable contact lens on keratometric indices and corneal thickness of keratoconus eyes examined with anterior segment optical coherence tomography

**DOI:** 10.1371/journal.pone.0270519

**Published:** 2022-07-08

**Authors:** Kaho Akiyama, Takashi Ono, Hitoha Ishii, Lily Wei Chen, Kohdai Kitamoto, Tetsuya Toyono, Junko Yoshida, Makoto Aihara, Takashi Miyai

**Affiliations:** 1 Department of Ophthalmology, The University of Tokyo Hospital, Bunkyo-ku, Tokyo, Japan; 2 Department of Ophthalmology, Graduate School of Medicine, University of Tokyo, Bunkyo-ku, Tokyo, Japan; 3 Department of Ophthalmology, International University of Health and Welfare Mita Hospital, Minato-ku, Tokyo, Japan; University of Toronto, CANADA

## Abstract

**Purpose/Aim:**

Detecting keratoconus (KC) progression helps determine the surgical indication for corneal cross-linking (CXL). This retrospective observational study aimed to examine changes in keratometric indices and corneal thickness in patients with KC who used rigid gas-permeable (RGP) contact lenses.

**Materials and methods:**

This study involved 31 eyes (31 patients) diagnosed with KC. No patient had used RGP or any other type of contact lenses for at least 1 month. Corneal topographic data were obtained using three-dimensional anterior segment optical coherence tomography before and after >1 month of RGP lens use.

**Results:**

The average and maximum keratometry values changed after using an RGP lens (-1.05 ± 1.92 D, p < 0.01 and -1.65 ± 4.20 D, p = 0.04, respectively); the spherical component of the anterior corneal surface became significantly smaller (p = 0.02). No change was observed in the central or thinnest corneal thickness values. Keratometric changes were greater in eyes with severe KC than in those with moderate KC (p = 0.014).

**Conclusions:**

Keratometry and spherical components of the anterior corneal surface values decreased after RGP lens use; keratometric changes were greater in eyes with severe KC than in those with moderate KC. Corneal progression indices, including corneal thickness, posterior keratometry, and irregular astigmatism values, mostly remained unchanged. It is important to consider these findings when evaluating corneal topography of KC and preparing CXL.

## Introduction

Keratoconus (KC) is a noninflammatory ectatic corneal disorder that can lead to decreased visual acuity owing to progressive corneal thinning, protrusion, and scarring [1, 2]. In the early stages of KC, when corneal irregular astigmatism is minor, visual acuity can be corrected with spectacles or a soft contact lens. However, as KC progresses, corneal irregular astigmatism increases, and alternative approaches are required [3]. Accordingly, rigid gas-permeable (RGP) contact lenses are used to improve visual acuity in patients with KC; the lenses correct irregular astigmatism and reduce aberrations by reshaping the ocular surface and tear film [4, 5]. These lenses concurrently improve visual quality and provide ultraviolet protection [5].

Since it was first described by Wallensak et al. [6], corneal cross-linking (CXL) has become an increasingly popular treatment for halting the progression of KC [7]. Surgical indications for CXL are based on KC progression characteristics and include the following [8]: (1) steepening of the anterior corneal surface, (2) steepening of the posterior corneal surface, and (3) thinning and/or increase in the rate of corneal thickness change from the periphery to the thinnest point. Consequently, corneal topography, which used to be the primary method of KC diagnosis, has been replaced by corneal tomography, including anterior segment optical coherence tomography (AS-OCT), which helps detect changes in the posterior surface of the cornea and corneal thickness [8].

In addition, the progression of KC is evaluated with AS-OCT indices such as maximum keratometry (K_max_), average keratometry (Avg K), and thinnest corneal thickness (TCT) values, among others [9]; however, these values may change after initiating RGP lens usage and affect the timing of treatment by masking the natural progression of the disease. Some previous studies have shown that the corneal shape may change during the use of RGP lenses, but few studies have reported corneal topographic changes in the non-wearing state after mid- or long-term usage [10, 11]. Studies that examined patients who were already using RGP lenses compared the characteristics of the “on” and “off” states; however, no study has reported on changes in corneal tomography when patients with KC first start using RGP lenses.

In general, patients with KC need to stop using RGP lenses before undergoing CXL, which, although inconvenient, remains necessary for the accurate evaluation of corneal topography. Understanding the effects of RGP lens use on KC progression indices measured with AS-OCT is paramount to achieving good patient outcomes.

Fourier harmonic analysis of corneal topographic data allows the decomposition of patient refractive data into four components, namely, the spherical component, regular astigmatism, asymmetry, and higher-order irregularity [12, 13]. This technique is clinically relevant for detecting and evaluating KC because it helps quantify the degree of irregular astigmatism that is not corrected with spectacles [14, 15]. However, few previous studies have used Fourier harmonic analysis to examine corneal topographic changes after the use of RGP lenses in patients with KC [10]. Thus, the current study aimed to evaluate KC progression and irregular astigmatism indices associated with RGP lens use in patients with KC.

## Materials and methods

This observational study followed the tenets of the Declaration of Helsinki for research involving human subjects, and the study protocol was approved by the Institutional Review Board of Research Ethics Committee at the University of Tokyo Hospital (Identifier: 2020006NI). The requirement for written informed consent was waived; participants were provided with the opportunity to opt out of the study.

### Patients

Patients diagnosed with KC and newly prescribed RGP lens use at the University of Tokyo Hospital (Tokyo, Japan) between January 2016 and September 2020 were included in this study. Patients with a history of corneal surgery, corneal collagen cross-linking, ophthalmological infectious, inflammatory diseases, or any type of contact lens use within the previous month, were excluded from this study. KC was diagnosed by multiple corneal specialists with AS-OCT to examine corneal topography. Corneal specialists diagnosed collaboratively and were not blinded to the diagnosis. RGP lenses were prescribed and used daily in the daytime, and the patients were examined again after 1 month. Patients were required to attend a follow-up visit wearing their lenses, and the follow-up topography examination was performed just after RGP lens removal. Data obtained from only one eye from each patient were used for the analysis.

### Examination items

Patients’ demographic characteristics and corneal topographic data were retrospectively extracted from their electronic medical records. We evaluated the K_max_ (D), Avg K (D), Ks (D), central corneal thickness (CCT) (μm), and TCT (μm) values as KC progression indices. The Ks and Avg K of the anterior and posterior corneal surfaces before and after RGP lens use were also examined. The K_max_, Avg K, Ks, CCT, TCT, Ks, and Avg K values of the anterior and posterior corneal surfaces and topographic data were evaluated using AS-OCT (SS-1000 CASIA or SS-2000 CASIA2; Tomey Corporation, Inc, Aichi, Japan). Corneal assessment using AS-OCT was performed while the patient was not wearing the RGP lens.

To evaluate corneal irregular astigmatism, Fourier harmonic analysis of corneal topographic data was performed as previously reported [13]. Axial refractive power data on the 6-mm Mire ring were decomposed into a series of trigonometric components as follows. Dioptric powers on a Mire ring I and Fi (σ) values were transformed into trigonometric components with the Fourier series harmonic analysis program included in CASIA or CASIA2:

$${\text{F}}_{\text{i}}\left(\sigma \right)=a_{0}+c_{1}\;\text{cos}\left(\sigma -\alpha_{1}\right)+c_{2}\;\text{cos}\;2\left(\sigma -\alpha_{2}\right)+c_{3}\;\text{cos}\;3\left(\sigma -\alpha_{3}\right)+\cdots +c_{n}\;\text{cos}\;n\left(\sigma -\alpha_{n}\right)$$

where a_0_ is the spherical component of the ring; 2c_1_ is the asymmetry (tilt or decentration) component; 2c_2_ is the regular astigmatism component; summations of c_3_…_n_ are the higher-order irregularity components. These calculations were performed on rings 2 to 9, which approximate the central 6-mm zone of the cornea, and the results were averaged for each parameter (spherical component, regular astigmatism, asymmetry, and higher-order irregularity).

The severity of KC was graded using the Amsler–Krumeich classification [16] and the Collaborative Longitudinal Evaluation of Keratoconus (CLEK) study group criteria [17]. Eyes were classified into two groups according to each criterion: moderate KC (stages 1 and 2) and severe KC (stages 3 and 4), according to the Amsler–Krumeich classification, and severe KC (Avg K of >52 D) and moderate KC (Avg K of ≤52 D), according to the CLEK criteria.

### Statistical analysis

All data were reported as the mean ± standard deviation unless otherwise specified. The normality of distribution was examined using the Shapiro–Wilk test. The paired t-test was performed to compare corneal topographic indices before and after RGP lens use. The unpaired t-test was performed to compare examination items between the severe and moderate KC groups. Statistical analysis was performed with Bell Curve for Excel (Social Survey Research Information, Tokyo, Japan). P-values of <0.05 were considered significant.

## Results

In total, 31 eyes of 31 patients were examined in this study. The mean age of the patients was 32.5 ± 14.6 years, and 24 (77%) patients were men. The Avg K, K_max_, and TCT values before the fitting of the RGP lens were 50.2 ± 7.2 D, 57.2 ± 11.7 D, and 442.9 ± 67.8 μm, respectively. Twelve eyes used a traditional spherical RGP lens, and 19 eyes used a customized multicurve RGP lens. According to the Amsler–Krumeich classification, 18 eyes had moderate KC (stage 1: 10 eyes, stage 2: 8 eyes), and 13 eyes had severe KC (stage 3: 6 eyes, stage 4: 7 eyes). According to the CLEK criteria, 23 eyes had moderate KC, and 8 eyes had severe KC.

### Corneal power and corneal thickness

**Fig 1:**
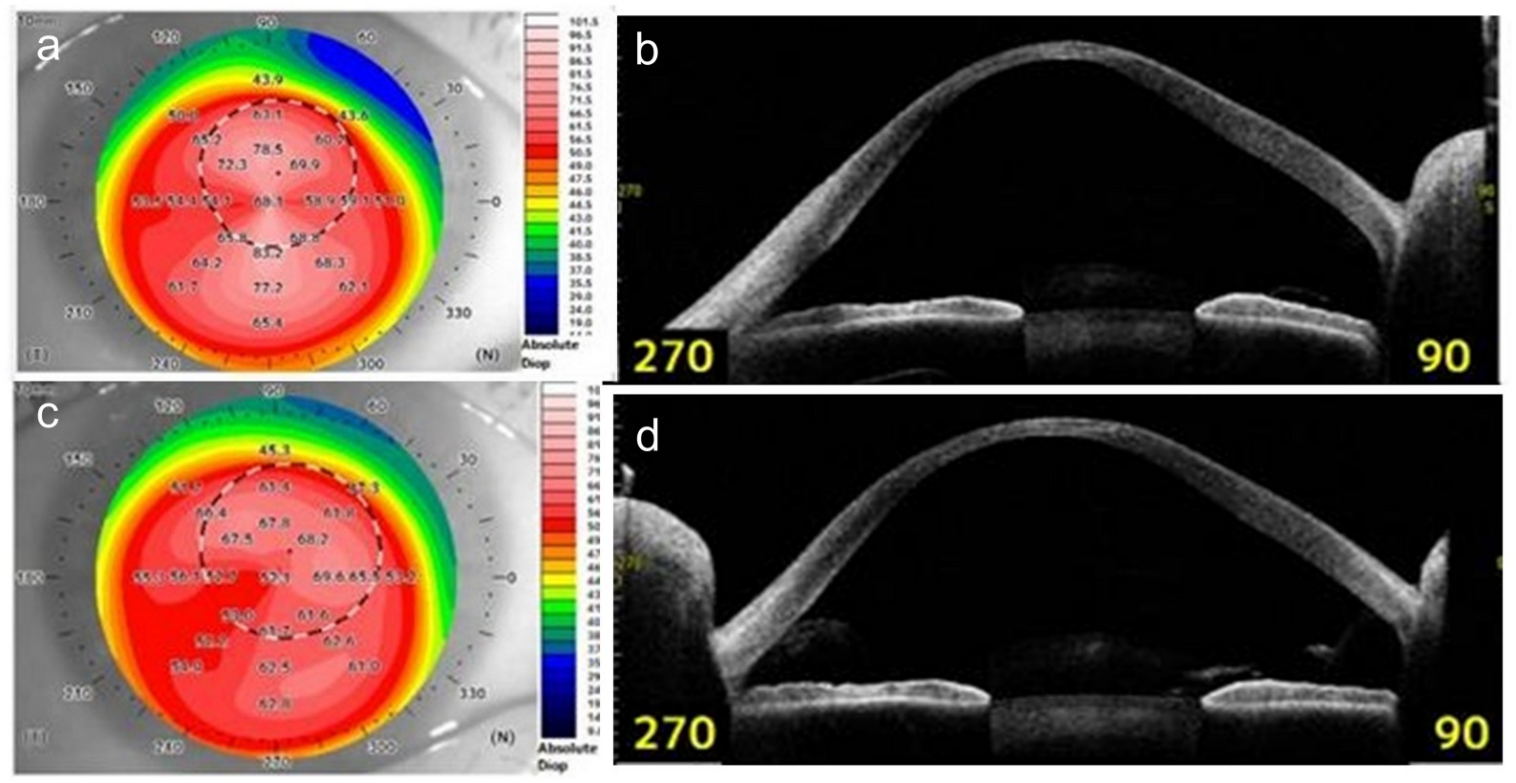
Representative corneal topographic maps and tomographic images of a patient with keratoconus. The maps and images were before and after using a rigid gas-permeable contact (RGP) lens. (A) Corneal topographic map before using the RGP lens. (B) Tomographic image of the cornea with anterior segment optical coherence tomography before using the RGP lens. (C) Corneal topographic map after using the RGP lens. The keratometric values appear to have decreased after RGP lens use compared with those before RGP lens use. (D) Tomographic image of the cornea with anterior segment optical coherence tomography after using the RGP lens. Corneal protrusion appears to have decreased after RGP lens use compared with that before RGP lens use.

Changes in K_max_, Avg K, keratometry at steep axes (Ks), CCT, and TCT values after RGP lens use are summarized in Table 1. There was a significant decrease in the K_max_ (-1.65 ± 4.20 D, p = 0.04), Avg K (-1.05 ± 1.92 D, p < 0.01), and Ks (-1.45 ± 3.22 D, p = 0.02) values after the patients started using the RGP lens. In contrast, there was no significant change in CCT or TCT values. There was a significant decrease in the Avg K and Ks values of the anterior corneal surface; however, there was no significant change in the corresponding values of the posterior corneal surface (Table 2). In addition, there was no significant difference in changes of Avg K, Ks, and K_max_ values between the spherical (N = 12) and multiple curve (N = 19) lens types. Representative changes in corneal topographic maps and tomographic images observed in a 30-year-old man with KC are shown in Fig 1. In this patient, the values of K_max_ decreased from 87.61 D to 69.58 D, Avg K decreased from 70.59 D to 63.22 D, and Ks decreased from 82.33 D to 65.33 D with the use of the RGP lens. CCT values remained at 290 μm, but TCT values changed from 261 μm to 256 μm.

**Table 1:** Changes in keratoconus progression indices before and after the use of a rigid gas-permeable contact lens.

	Before	After	Change	p-value
**K**_**max**_ **(D)**	57.2 ± 11.7	55.6 ± 9.5	-1.65 ± 4.20	0.04*
**Avg K (D)**	50.2 ± 7.2	49.2 ± 5.9	-1.05 ± 1.92	<0.01*
**Ks (D)**	52.4 ± 8.5	50.9 ± 6.4	-1.45 ± 3.22	0.02*
**CCT (μm)**	465.3 ± 53.3	468.5 ± 56.1	3.23 ± 21.0	0.40
**TCT (μm)**	435.9 ± 54.7	429.7 ± 64.7	-6.16 ± 23.5	0.15

*: p-value <0.05, paired t-test.

All data are expressed as the mean ± standard deviation. RGP, rigid gas-permeable; K_max_, maximum keratometry; Avg K, average keratometry; Ks, keratometry at steep axes; CCT, central corneal thickness; TCT, thinnest corneal thickness.

**Table 2:** Changes in keratoconus progression indices of the anterior and posterior corneal surface before and after the use of a rigid gas-permeable contact lens.

Anterior surface	Before	After	Change	p-value
**Avg K (D)**	55.9 ± 8.0	54.8 ± 6.5	-1.17 ± 2.14	<0.01*
**Ks (D)**	58.4 ± 9.4	56.7 ± 7.2	-1.62 ± 3.58	0.02*
**Posterior surface**				
**Avg K (D)**	-7.6 ± 1.4	-7.5 ± 1.2	0.10 ± 0.37	0.13
**Ks (D)**	-8.0 ± 1.6	-7.8 ± 1.3	0.13 ± 0.45	0.11

*: p-values <0.05, paired t-test.

All data are expressed as the mean ± standard deviation. RGP, rigid gas-permeable; Avg K, average keratometry; Ks, keratometry at steep axes.

### Fourier harmonic analysis of the anterior and posterior corneal surfaces

Changes in corneal parameters evaluated with Fourier harmonic analysis for the anterior and posterior corneal surfaces are summarized in Table 3. The spherical component of the anterior corneal surface significantly decreased after the use of the RGP lens (p = 0.02). There was no significant change in the values of regular astigmatism, asymmetry component, or high-order irregularity of the anterior corneal surface. Finally, Fourier harmonic analysis showed no significant change in the values of the four parameters of the posterior corneal surface.

**Table 3:** Changes in corneal anterior and posterior surface indices evaluated with Fourier harmonic analysis before and after the use of a rigid gas-permeable contact lens.

Anterior corneal surface	Before	After	Change	p-value
**Spherical component**	55.1 ± 7.2	54.2 ± 6.0	-0.85 ± 1.93	0.02*
**Regular astigmatism**	2.5 ± 2.3	2.0 ± 1.1	-0.53 ± 1.94	0.14
**Asymmetry component**	4.9 ± 3.3	4.6 ± 3.3	-0.33 ± 1.57	0.25
**Higher order irregularity**	0.6 ± 0.4	0.5 ± 0.4	-0.03 ± 0.21	0.49
**Posterior corneal surface**				
**Spherical component**	-7.2 ± 1.1	-7.1 ± 1.0	0.08 ± 0.30	0.15
**Regular astigmatism**	0.3 ± 0.3	0.3 ± 0.2	-0.02 ± 0.19	0.57
**Asymmetry component**	1.3 ± 0.7	1.3 ± 0.7	-0.01 ± 0.17	0.85
**Higher order irregularity**	0.1 ± 0.0	0.1 ± 0.1	0.01 ± 0.05	0.23

*: p-value <0.05, paired t-test.

All data are expressed as the mean ± standard deviation.

### Comparison of changes in corneal indices based on the severity of KC

**Fig 2:**
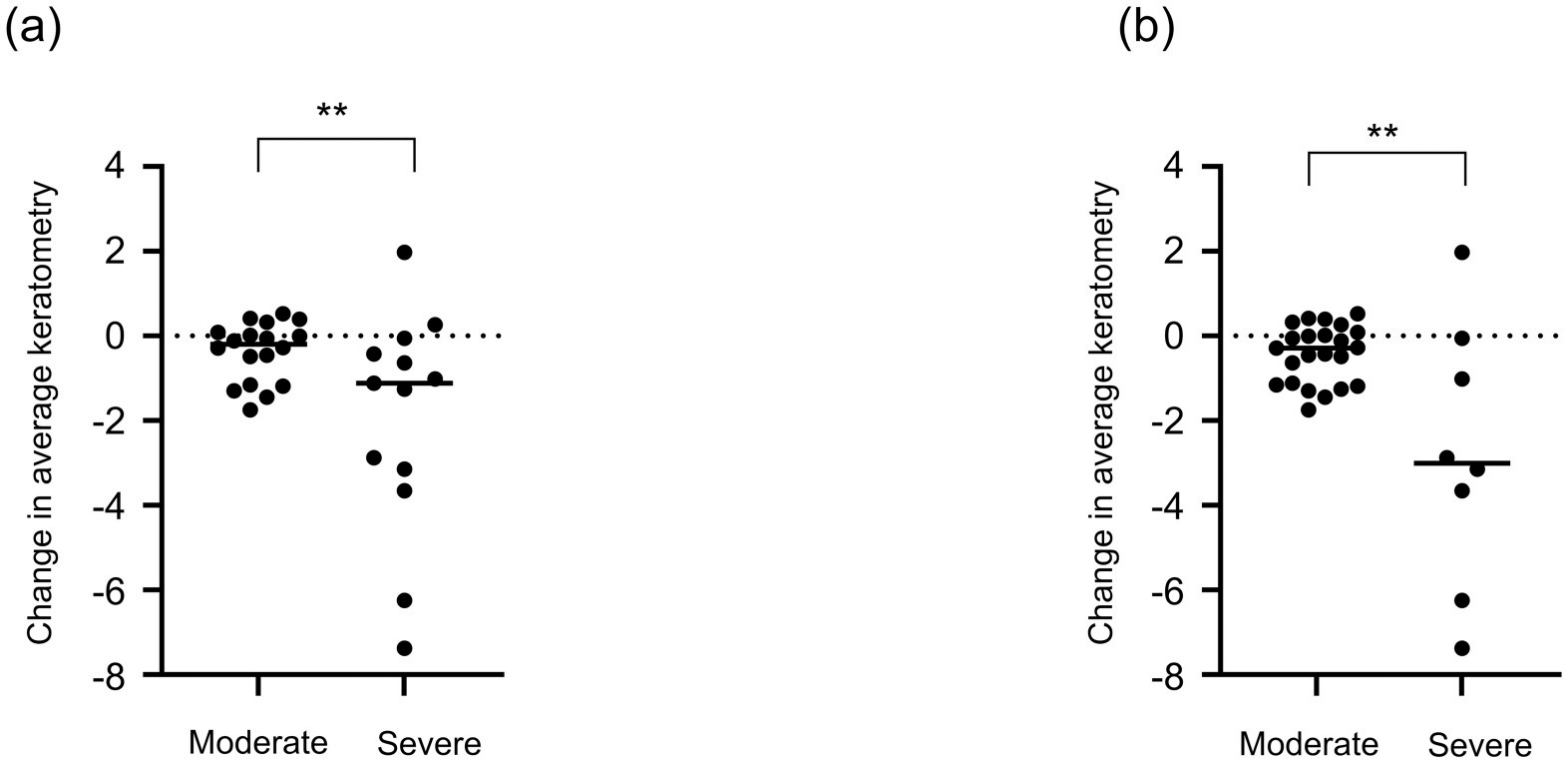
Changes in average keratometric values in patients with keratoconus using a RGP lens. (A) Comparison of keratometric changes during RGP lens use in eyes with severe and moderate KC based on the Amsler–Krumeich classification. The average keratometric value appears to have decreased in the severe KC group compared with that in the moderate KC group (p < 0.05, unpaired t-test). (B) Comparison of keratometric changes during RGP lens use in eyes with severe and moderate KC based on the Collaborative Longitudinal Evaluation of Keratoconus study group’s criteria. The average keratometric value appears to have significantly decreased in the severe KC group compared with that in the moderate KC group (p < 0.05, unpaired t-test). RGP, rigid gas-permeable contact, KC, keratoconus.

There was a significant decrease in the Avg K values after RGP lens use in both the severe and moderate KC groups, according to the Amsler–Krumeich classification (Table 4) and CLEK criteria (Table 5). In intragroup comparisons, the severe KC group showed a significantly greater change in Avg K values than the moderate KC group, according to both the Amsler–Krumeich classification (p = 0.02) and CLEK criteria (p = 0.014) (Fig 2). There was no significant difference in other parameters.

**Table 4:** Changes in keratoconus progression indices of severe and moderate keratoconus based on the Amsler–Krumeich classification before and after the use of a rigid gas-permeable contact lens.

Severe KC	Before	After	Change	p-value
**K**_**max**_ **(D)**	65.5 ± 12.6	62.6 ± 9.90	-2.89 ± 5.93	0.10
**Avg K (D)**	55.6 ± 7.97	53.6 ± 6.26	-1.97 ± 2.63	0.02*
**CCT (μm)**	433.3 ± 64.0	437.5 ± 65.9	4.15 ± 28.8	0.61
**TCT (μm)**	395.4 ± 54.3	383.6 ± 67.4	-11.8 ± 35.3	0.25
**Moderate KC**				
**K**_**max**_ **(D)**	51.2 ± 4.83	50.5 ± 5.46	-0.75 ± 2.07	0.14
**Avg K (D)**	46.3 ± 2.73	45.9 ± 2.78	-0.38 ± 0.70	0.04*
**CCT (μm)**	488.4 ± 26.1	490.9 ± 32.3	2.56 ± 14.0	0.45
**TCT (μm)**	465.1 ± 28.2	463.0 ± 28.5	-2.11 ± 7.19	0.23

*: p-values <0.05, paired t-test.

All data are expressed as the mean ± standard deviation. KC, keratoconus, K_max_, maximum keratometry; Avg K, average keratometry; CCT, central corneal thickness; TCT, thinnest corneal thickness.

**Table 5:** Changes in keratoconus progression indices of severe and moderate keratoconus based on the Collaborative Longitudinal Evaluation of Keratoconus study group’s criteria before and after the use of a rigid gas-permeable contact lens.

Severe KC	Before	After	Change	p-value
**K**_**max**_ **(D)**	72.0 ± 10.6	68.0 ± 6.89	-4.00 ± 7.32	0.17
**Avg K (D)**	60.3 ± 6.20	57.5 ± 4.22	-2.80 ± 3.10	0.04*
**CCT (μm)**	407.1 ± 66.8	409.5 ± 66.3	2.38 ± 36.4	0.86
**TCT (μm)**	371.3 ± 51.7	352.3 ± 62.4	-19.0 ± 42.9	0.25
**Moderate KC**				
**K**_**max**_ **(D)**	52.1 ± 5.70	51.3 ± 6.08	-0.83 ± 2.07	0.17
**Avg K (D)**	46.7 ± 2.78	46.3 ± 2.80	-0.43 ± 0.68	0.01*
**CCT (μm)**	485.5 ± 26.9	489.0 ± 32.5	3.52 ± 13.4	0.22
**TCT (μm)**	458.3 ± 31.5	456.7 ± 33.9	-1.70 ± 9.13	0.38

*: p-values <0.05, paired t-test.

All data are expressed as the mean ± standard deviation. KC, keratoconus, K_max_, maximum keratometry; Avg K, average keratometry; CCT, central corneal thickness; TCT, thinnest corneal thickness

## Discussion

In the present study, Avg K and Ks values significantly decreased 1 month after RGP lens use in patients with KC, while CCT and TCT values did not change. As tomographic analyses revealed that these changes were observed only on the anterior corneal surface, these findings suggest that RGP lens use may clinically affect the anterior corneal surface and change the corneal curvature. A previous study reported that suspending an RGP lens use for a week significantly increased keratometric power [11]. Studies on orthokeratology have shown that contact lenses may induce corneal warpage owing to remodelling of the anterior corneal layers, particularly the corneal epithelium [18, 19]. The present study findings support those of previous studies on the flattening of corneal topography resulting from RGP lens use.

Furthermore, Fourier harmonic analysis revealed that RGP lens use for 1 month changed the value of the spherical component of the anterior cornea, which consisted of the corneal epithelium and stroma. Evidence is limited on corneal warpage resulting from contact lens use and the associated biomechanism; thus, the present findings may help elucidate the biomechanism of corneal changes during the use of RGP lenses. Fourier harmonic analysis has helped detect subclinical KC [14]. Koh *et al*. examined refractive changes in the anterior and posterior cornea using this approach and suggested that the spherical component, regular astigmatism, and asymmetry component values of the posterior cornea decreased during the use of an RGP lens in the same eye [10]. In contrast to their study, herein, the values of topographic parameters in eyes without RGP lens use were examined. The results indicated that the effect of RGP lens usage on corneal topography should not be ignored. It is necessary to consider the effect of RGP lens use on corneal topography in evaluating the progression of KC and considering the indication of CXL. Our results suggested that as posterior keratometric values and corneal thickness did not change after RGP lens use in our study, these indices might be useful in evaluating the progression of KC in patients with RGP lens.

Three approaches to RGP lens use in KC have been described, namely apical touch, three-point touch, and apical clearance [20]. In apical touch fitting, the lens rests on the corneal apex. In three-point touch fitting, the lens lightly touches the corneal apex and rests on the mid-peripheral area of the cornea. In apical clearance fitting, the lens rests on the mid-peripheral area of the cornea and does not touch the corneal apex. Apical touch fitting and three-point touch fitting are preferred in KC because the lens touches the corneal apex, causes corneal flattening, and improves irregular astigmatism; by contrast, apical clearance causes corneal steeping [21]. Romero-Jiménez *et al*. reported that wearing an RGP lens for 14 days flattened the anterior cornea and that apical touch fitting caused greater corneal flattening than three-point touch fitting [22]. We did not evaluate the impact of RGP lens fitting approaches because of the lack of relevant data. Different approaches to the corneal fitting of an RGP lens may have different effects on corneal topography; future prospective studies are required to elucidate the impact of RGP lens types and approaches to fitting on patient outcomes. Additionally, to examine time-serial changes, keratometric data should be evaluated each week after removing the contact lens.

The type of RGP lens was considered in this study, but there was no significant difference in the change of Kmax, Ks, and AvgK. Different lens fitting occurs between traditional and customized lenses. A traditional RGP lens tends to fit with apical fitting in KC eyes due to the corneal steepening and sometimes has some problems regarding lens stabilization and risk of epitheliopathy. In contrast, multicurve customized lenses were used to overcome this problem and were generally used with three-point touch [23]. Because these lenses characteristics could lead to a difference in mechanical stress and change in the cornea, lens fitting information should be examined in the future.

This study had some limitations. First, the progression of KC during the observation period was not considered. Only patients who did not require CXL or corneal transplantation were treated with an RGP lens. Furthermore, we evaluated topographic changes 1 month after RGP lens use, and KC progression was unlikely to be rapid in this context. Second, owing to the retrospective nature of the present study, the materials and oxygen transmissibility (Dk/t) values of the RGP lens differed, while the observation period was limited to 1 month. The long-term effects of RGP lens use on corneal surface parameters may need to be evaluated in future multicenter prospective randomized controlled trials. Furthermore, clinical examinations using only AS-OCT were performed. However, other evaluating devices, such as Scheimpflug devices (Pentacam, Oculus GmbH, Wetzlar, Germany), could quantitatively evaluate corneal topographic changes. Further clinical research based on Scheimpflug devices utilizing the Belin ABCD classification/staging system [24, 25] is also required. Finally, the device repeatability for patients with KC might need to be considered, since AS-OCT was performed on each patient only once in the current study. However, future research with multiple examinations, more than three times, at each examination point would provide more accurate results according to the report of Brunner et al [26].

In conclusion, keratometry and spherical component values of the anterior corneal surface significantly decreased after the use of an RGP lens, and changes in keratometric values were greater in eyes with severe KC than in those with moderate KC. Corneal progression indices related to corneal thickness and posterior keratometry, and irregular astigmatism remained mostly unaffected. These findings may help predict keratometric changes in patients required to suspend RGP lens use to prepare for corneal treatment.

## Supporting information

S1 Dataset (XLSX)
